# Matrix-assisted laser desorption/ionisation mass spectrometry imaging and its development for plant protein imaging

**DOI:** 10.1186/1746-4811-7-21

**Published:** 2011-07-05

**Authors:** Julia Grassl, Nicolas L Taylor, A Harvey Millar

**Affiliations:** 1 ARC Centre of Excellence in Plant Energy Biology and Centre for Comparative Analysis of Biomolecular Networks, M316, The University of Western Australia, Crawley, WA 6009, Australia

**Keywords:** MALDI MS imaging, MSI, mass spectrometry imaging, plant, plant imaging soybean, protein, protein imaging

## Abstract

Matrix-Assisted Laser Desorption/Ionisation (MALDI) mass spectrometry imaging (MSI) uses the power of high mass resolution time of flight (ToF) mass spectrometry coupled to the raster of lasers shots across the cut surface of tissues to provide new insights into the spatial distribution of biomolecules within biological tissues. The history of this technique in animals and plants is considered and the potential for analysis of proteins by this technique in plants is discussed. Protein biomarker identification from MALDI-MSI is a challenge and a number of different approaches to address this bottleneck are discussed. The technical considerations needed for MALDI-MSI are reviewed and these are presented alongside examples from our own work and a protocol for MALDI-MSI of proteins in plant samples.

## Introduction

Knowledge of plant development and function can be obtained by determining the distribution of proteins and metabolic processes within plant tissues. The differentiation of leaf, stem, root and floral architecture from the germinating seed provides an excellent example of the changes in distribution of proteins and metabolic processes. In addition protein abundance differences are also apparent in cell types within a tissue section. Matrix-Assisted Laser Desorption/Ionisation mass spectrometry imaging (MALDI-MSI) has the potential to provide new insights into the molecular analysis of plants by providing high spatial resolution information about proteins and potentially quantitative changes during plant development or those induced by environmental variation. In medical biology, MALDI-MSI of proteins has already begun a revolution in diagnostic immuno-histochemistry (IHC) by providing new disease biomarkers [1-3]. To date the literature of MALDI-MS imaging in plants is limited to mostly small molecules such as metabolites and lipids. The development of techniques for assessing the spatial localisation of plant proteins will differ from mammalian research because the diagnostics-driven focus on biomarkers in medical biology is largely absent in plant research. Moreover, once routine quantitation protocols are developed, these will likely provide a new focus for biomarkers in plant breeding and plant disease diagnosis. Here we review the technical MALDI-MSI literature including animal and human disease, the emerging literature in plants, and provide examples and current protocols for MALDI-imaging of proteins in plant tissue from our own research. A protocol for MALDI-MS imaging using plant tissue is available as additional file 1.

## Development of MALDI-MS imaging

MALDI-MSI was first reported in 1994 [4] and has been applied to visualise peptides and proteins since 1997 [5]. MALDI-MSI has since become a powerful technique that enables the identification and localisation of biological compounds directly on tissue surfaces. The predominate method used for imaging has been MALDI-ToF mass spectrometry (MS), however FT-ICR, ion-trap and Q-ToF have also been used for MSI of small molecules. MALDI-MSI has been used to image the distribution of a wide range of compounds, including proteins, lipids, pharmaceuticals and metabolites. In recent years, it has provided biomarkers in tissue samples that can be used to identify cancerous regions [2,6-8], as well as define tumour margins [9] and to monitor drug metabolism in various organs [10]. The ability to determine the distribution of peptides and proteins in cells of animals is making MALDI-MSI a valuable tool to understand underlying biological processes [11]. Increasingly MALDI-MSI has direct applications in cancer diagnostics and treatment; new paradigms in boundaries for tissue removal for future samples have been set. The decision where to set the tumour margin; weighing up the chance of leaving some cancerous cells behind or to remove too much tissue, and potentially causing some unnecessary harm to the patient, may be made easier [9,12]. The advantage of MSI over IHC is that IHC is a targeted approach, whereas MSI is not. Conversely the advantage of IHC over MSI is sensitivity and no restriction in protein size. Both have an important place in pathology laboratories. More broadly and in a range of species, MALDI-MSI has allowed the simultaneous analysis of the distribution of hundreds of peptides and proteins directly from a tissue section, which is particularly valuable when a lack of antibodies precludes protein identifications by IHC such as in the case of most plants.

## Progress in plant MALDI MS imaging to date

In plants, a range of reports have used MALDI-MSI to assess the spatial distribution of sugars, metabolites and lipids. There are reports where surface molecules such as epicuticular lipids, waxes and also secondary metabolites, such as flavonoids or alkanes, were measured on the surface of *Arabidopsis thaliana *flowers, leaves and roots [13-16]. Cha *et al*. [14] used colloidal silver laser desorption/ionization mass spectrometry to directly profile and image epicuticular waxes on leaves and flowers from *Arabidopsis thaliana*. One example of MSI in soybean was reported for determining the presence of agrochemical compounds (herbicides or insecticides) on the leaf surface [17]. Other reports have considered the intracellular spatial distribution of metabolites from plant tissue sections [18-20]. Goto-Inoue *et al*. [21] recently showed the spatial distribution of gamma-aminobutyric acid (GABA) in the seed of aubergines and this metabolite was identified by comparison to a synthesised standard. Ng *et al*. [22] demonstrated the spatial profiling of the phytochemicals and secondary metabolites by direct analysis of plant tissue using MALDI-MSI. These results clearly differentiated the relative abundance of metabolites in different tissue regions including the cortex, phloem, xylem, rim and pith. MALDI-MSI has also been used to reveal a spatial distribution of lysophosphatidylcholine and phospatidylcholine in rice endosperm and bran respectively, whereas α-tocopherol was only present in the rice germ [23]. The localisation of primary metabolites such as sucrose, glucose-6-phosphate and arginine in wheat seed [19] and potatoes [23] has been mapped using MALDI-MSI. The identity of these metabolites could not be derived using MS/MS methods, however synthesised standards of these molecules were available for comparison. In 1997, Stahl *et al*. [24] applied MALDI-MS and high-performance anion-exchange chromatography to analyse two high molecular weight fructans from *Dahlia variabilis *L. and also carried out direct tissue analysis on the epidermal and parenchymal tissue of onion bulbs (*Allium cepa L*.). *Allium cepa L*. contains various isomeric fructans and more than 50 compounds were detected from both techniques with masses ranging from < 2000 to 10 000 Da. This was one of the first recorded applications of direct tissue analysis using a MALDI-ToF instrument. Other sugars have been mapped on wheat stems using MALDI-MSI [25] and the ions were identified using LC-MS/MS.

The use of atmospheric pressure infrared MALDI-MSI using a Q-ToF instrument has allowed imaging of a large number of lipids and metabolites in a number of plant organs and species [18]. Here more than 50 metabolites and various lipids were detected in different plant tissues including flowers of white lily, (*Lilium candidum*), fruits (banana, *Musa paradisiacal*; strawberry, *Fragaria ananassa*; tomato, *Solanum lycopersicum*), leaves (coriander, *Coriandrum sativum*; peace lily, *Spathiphyllium*), tubers (potato, *Solanum tuberosum*), bulbs (onion, *Allium cepa*; garlic, *Allium sativum*), and seeds (almond, *Prunus amygdalus*). In a more recent publication Hamm *et al*. [26] used grapevine leaves and revealed specific locations of *Plasmopara viticola *pathogen infection. Using matrix-free MSI, matrix-related problems could be eliminated and with single cell resolution the spatial distribution of secondary metabolites, such as flavonoids, were detected in fresh and cryo-sectioned plant tissue of *Arabidopsis thaliana *and *Hypericum *leaves and flowers [27]. More recently matrix-free MSI utilizing an infrared laser for orthogonal time-of flight MS was used to identify metabolites and other low molecular weight ions in tobacco (*Nicotiana tabacum*) leaves infected with *Phytophtora nicotianae *[28].

There have been very few reports of MALDI-MSI for proteins in plants. A single protein, the lipid transfer protein Pru p3, has been detected by MALDI-MSI in the peel of the peach fruit [29]. This protein identification was determined using high resolution MS at the protein level and tandem MS measurements of proteolytic digests. In a recent review by Kaspar *et al*. [30], discussing the progress in the field of MALDI-MSI on small molecules, discriminative peptides in barley grain sections were highlighted as examples.

## Mass Spectrometers

A number of different ionization techniques such as desorption electrospray ionization (DESI) [31], secondary ion mass spectrometry (SIMS) [32], and matrix-free and matrix-assisted laser desorption/ionization (MALDI), have been investigated. Recent and past instrumentation for MSI are described by the MALDI-MSI Interest Group http://www.maldi-msi.org. Using the DESI ionization technique, molecules are ionized without addition of organic matrix by electrospray. DESI allows for direct analysis [33] and imaging [34] of biological tissues and other surfaces [35]. However, DESI has a limited spatial resolution of 0.3-0.5 mm, which allows profiling but is not sufficient for high resolution imaging. Both MALDI-ToF and ToF-SIMS instruments can provide submicrometer spatial resolution and each have advantages in imaging mass spectrometry. There are several published articles discussing and comparing MALDI and SIMS imaging [36,37]. For protein imaging MALDI is the most used form of ionisation, coupled to a wide range of different mass analysers, including ToF, ToF-ToF, QqToF, ion-trap (both linear and spherical) and Fourier transform ICR (FT-ICR). Each of these has their own merits and this has been discussed and reviewed previously [38]. This review focuses on MALDI-ToF MS imaging as the majority of data have been obtained with this platform and it is currently the most widely distributed instrument for user access.

## Tissue handling and sample preparation

Spatial resolution in MALDI-MSI depends on sample preparation protocols, the crystal structure of applied matrices and the mass resolution of the laser ablation [5]. The latest commercial MALDI-ToF mass spectrometers can obtain < 20 μm pixel resolution. However in most cases this is not achievable as the sensitivity suffers in very small sample areas. Furthermore, at high spatial resolution the crystal size of the matrix may be larger than the expected resolution and thus become the limiting factor.

**Figure 1:**
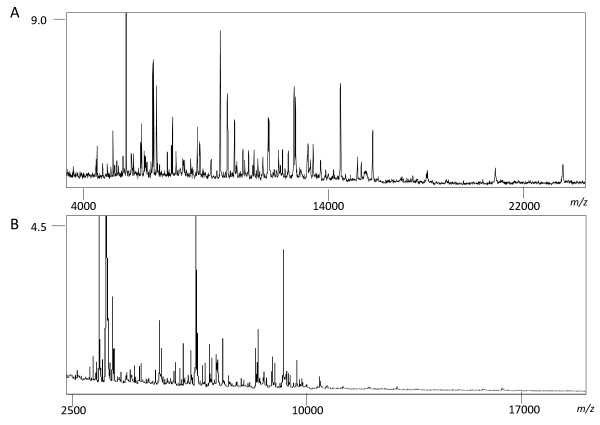
**Average mass spectra across a tissue cross-section of a mouse liver (A) and a soybean cotyledon (B)**.

Spatial ion distribution analysis with MALDI-MSI requires a sample preparation protocol that avoids the removal and relocation of molecules to ensure that the measured distributions reflect the distribution of the living biological system. While imaging of metabolite and lipids have been analysed successfully in both animals and plants (250-1,000 Da), MALDI-MSI of higher mass proteins (2,000-25,000 Da) has only been widely implemented in animal tissue. Ionization of intact proteins from plant cross-sections has presented special challenges. Tissue sections from mouse liver and soybean cotyledons were analysed using sinapinic acid (SA) as a matrix by MALDI-MSI. Comparison of the average spectra show that plant samples have relatively little ionisation in the > 15,000 range while more ions are apparent in protein rich animal tissues (Figure 1).

**Figure 2:**
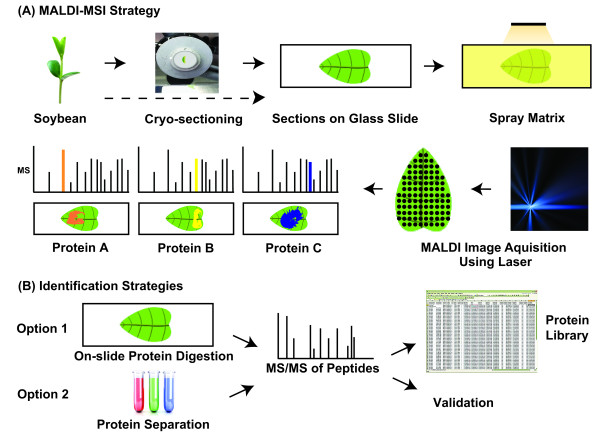
**Representative diagram of the MALDI-MSI strategy**. (A) The epidermis of whole leaves can be sprayed with a matrix directly or the tissue is cut into 10-15 μm sections and the sections are coated with a matrix on glass slides. The data are acquired across the section in the form of a raster of predefined resolution, and a mass spectrum of each raster-point is recorded. For each *m/z *value the ion intensity and distribution is collected. (B) Two options for identification of potential biomarkers are proposed. In some case, the slide may be coated with trypsin for on-slide protein digestion and peptides are analysed as above using reflectron mode and MS/MS. More commonly, the tissue region of interest needs to be excised and separated, isolating the *m/z *ion of interest. The ion of interest can then be digested with trypsin and analysed using LC-MS/MS.

Sample handling and preparation are crucial to obtain good quality images in a reproducible manner [39]. The type of freezing and preservation of tissue is critical for preparing optimal sections for MALDI-MSI. For IHC the use of propane as a coolant is optimal, however only tissue samples of a few millimetres can be frozen as larger samples break and show cracks in the tissue section [40]. The same breaking and cracking using liquid nitrogen also occurs in plant sections, hence a more gentle freezing method was required. Using a steel stage frozen in dry-ice, the tissue can be placed on the frozen metal until completely frozen (Figure 2A). Moreover embedding plant tissue in gelatine could not prevent freeze-induced cracking damage when using liquid nitrogen.

During cryo-sectioning the interior of frozen tissue samples are exposed. Embedding in optimal-cutting temperature (OCT) or another polymer-based embedding material can contaminate the section as the blade smears over the tissue and should be avoided as this interferes with the ionisation process [36,39]. Embedding in gelatine [41] and agarose [42] has been used for fragile samples, but mostly sections are cut directly on the frozen tissue. In contrasts to the compact, liquid filled structure of most animal tissues, plant cells have rigid cell walls and abundant air-spaces. In order to avoid tearing of the sections, the use of embedding materials is very common in plant microscopy [43,44]. In our hands frozen sections of plant tissue sections without the aid of an embedding material showed the greatest spectral quality. However embedding in gelatine showed an improvement in localisation, lateral resolution and reproducibility, with some loss in signal-to-noise. A recent paper has shown the use of carboxymethyl cellulose sodium (CMC) as an embedding method for rice grains [45]. Here rice grains were sectioned to differentiate metabolites in regions such as the endosperm, germ and bran. The use of CMC and the use of adhesive tape has enabled sectioning of hard tissue from plants, whole animals, as well as teeth and bone [40]. Interestingly we have observed that soaking tissues in sucrose improves the lateral resolution during imaging. The displacement of water with sucrose and filling of air spaces between cells dramatically improves cryosectioning and minimised de-localisation and the loss of signal [46]. The optimal cutting temperature of the cryostat is important and dependent on the tissue type, higher (-15°C) temperatures makes cutting easier however it can produce ice crystals, lower temperatures (-20°C) provide better sections (for a review refer to Kawamoto [40]).

Washing sections after cutting can also improve imaging. Seeley *et al*. [47] and Lemaire *et al*. [48] tested and discussed a range of organic solvents for removal of salts and contaminants to improve spectra quality. For protein imaging, the use of 2-propanol has been shown to be superior [47]. For lipids on the other hand the use of xylene or other organic treatments could be beneficial [48]. We observed better results with ice-cold 2-propanol. Washing with water had negative effects due to the large number of soluble proteins in plant tissues. Seeley *et al*. [47] further observed that immediate washing of the section allows longer storage of the slides before matrix deposition and analysis.

In summary, plant tissues used for analysis should be prepared and frozen using dry ice immediately after collection to preserve morphology and minimize protein degradation through proteolysis. The use of liquid nitrogen should be avoided for plant tissue as the high water content results in large crystal formation when the tissue is frozen too rapidly to such low temperature. The tissue can then be sectioned in a cryostat to give 10- to 15-μm-thick sections which are thaw-mounted onto microscope slides, pre-coated with an electrically conductive material (Figure 2A). Other sample plates such as gold-coated or stainless steel metal plates can also be used. In order to increase peak intensity the tissue can be washed with an organic solvent such as ethanol or 2-propanol acting as a fixative and to wash off lipids and salts and the use of xylene has been shown beneficial for lipid samples [48]. Complete drying of the tissue sections is important in order to preserve protein localisation.

## Matrix choice for MALDi-MS imaging of proteins

In 1987, Hillenkamp and co-workers [49] discovered that molecular ion species can be produced from large proteins by laser desorption, without much fragmentation, if these molecules are mixed with small organic compounds that serve as matrices. The requirements of a matrix are that it has a strong absorbance at the laser wavelength and is capable of sublimation [50]. The typical preparation protocol for protein analysis by MALDI-ToF MS is to mix or cover the sample with a matrix solution that contains small organic compounds such as α-cyano-4-hydroxycinnamic acid (CHCA) or sinapinic acid (SA). After the matrix crystallises, the sample plate is analysed inside the MALDI-ToF mass analyser. During the analysis process, the matrix material strongly absorbs the laser energy and quickly becomes vaporised. The analyte is embedded in the matrix and carried along in the fast vaporisation process. The molecules pick up a charge and travel down the time-of-flight tube, where they are analysed on basis of their *m/z *ratio (Figure 2A).

Sinapinic acid is the matrix of choice for MALDI-MSI of proteins in tissue sections [42,51,52]. For peptides and lipids 2,5-dihydroxybenzoic acid (DHB) or CHCA are better suited [51]. The choice of matrices for analysis of metabolites is more complex, primarily because using standard matrices, matrix ions often crowd the low-mass range, limiting confident detection of analyte ions of < 750 Da. Some excellent reviews covering the development of small-molecule imaging by using MALDI are available [53,54]. CHCA forms smaller crystals and hence produces a more homogeneous layer, allowing higher lateral resolution. DHB on the other hand, produces data with better signal-to-noise ratio, however, the large crystals produce a ring-like layer and cause non-homogeneous ionisation across the spot or section [55]. Using SA as a matrix, good reproducibility in ion intensity as well as spatial resolution was observed when comparing sequential sections of plant tissues.

More recently the application of CHCA mixed with aniline (ANI), which is basic, has been described to produce an ionic matrix [55]. In some cases this appears to produce optimal signal-to-noise intensities with the highest lateral resolution. More ionic matrixes have been tested by Lemaire *et al*. [55] and more recently ANI has been applied in lipid analysis [56] and whole tissue sample analysis [57]. In our hands, for MALDI-MSI of proteins in plant tissue sections, SA was the optimal matrix. DHB and CHCA both produced good signal to noise spectra for peptides in plant tissues, however, the protein localisations were not as specific for DHB. Furthermore, CHCA has been reported to produce more multiply charged ions than SA [52], which may be unfavourable for MALDI-MSI.

## Matrix deposition

For high-resolution imaging a homogeneous layer of the matrix solution should be applied to avoid formation of large crystals. Furthermore to avoid significant lateral re-localisation of analytes, the matrix should not be applied too wet. This can be achieved by using either a spotted array or a homogenous spray coating [39]. A continuous and homogenous matrix sprayed as layered coats allows the highest spatial resolution, however tightly spotted arrays yield higher reproducibility and often better spectral quality. Heterogeneous matrix application gives rise to random crystal formation, producing poor, mottled images with areas of high and low signal-to-noise ratios. A variety of matrix spotting devices are commercially available including acoustic [58], piezoelectric [11] or ink-jet printers [59]. A homogeneous layer of matrix can also be applied by coating the section with dry matrix using a paintbrush or similar [60]. For spray coating, a manual atomiser can be used [61] and numerous devices are also commercially available utilising oscillating capillary nebulisers [62] or vibrational volatilisation [62]. Spray coating and spotting has been shown to be most suitable for protein profiling on tissue sections [63], although a number of studies studying epicuticular small molecules in plants have used dry coating, especially using metal-based powder matrices [13-15,26]. We have used mist nebulising, using an ImagePrep (Bruker Daltonics), which applies several homogenous thin layers of matrix with optimal drying time (Figure 2). The experimental protocol used is available as a "Beginner's Guide" in the additional file 1.

## Mass spectrometry imaging

**Figure 3:**
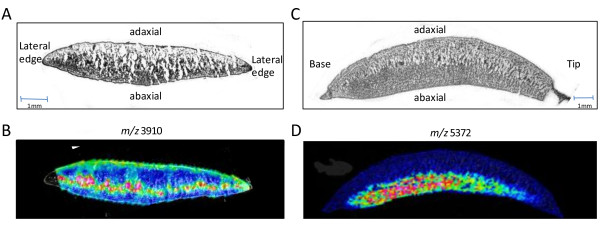
**MALDI-imaging in soybean cotyledons**. (A) A cross section of the cotyledon, cut across the cotyledon exposing the centre midway between the tip and the base. (B) MALDI-MSI cross section showing the peak intensity and localisation for *m/z *3918. (C) Cross section of the cotyledon cut axially from tip to base. (D) MALDI-MSI cross section showing the peak intensity and localisation for *m/z *5372.

There are two main experimental approaches to the use of MALDI-ToF MSI. For the first approach, pre-defined areas of the tissue sections are spotted with matrix and analysed; the protein profiles are compared using alignment software. This analysis may be performed at lower-resolution (> 500 μm) in order to make comparisons between representative areas on pieces of tissue, such as between two different morphological regions or between regions that respond to treatment or developmental changes. Thus, in this mode, fine spatial resolution is not required and the technique is commonly referred to as 'profiling'. On the other hand, for higher-spatial-resolution, the entire tissue section is analyzed through an ordered raster. Matrix is applied evenly across the section and spectra are acquired at intervals that define the image resolution, typically 30-100 μm in both the *x *and the *y *directions (Figure 2A). The resulting images allow rapid evaluation of protein localization differences across samples. As examples, localisation of molecular ions in a vascular tissue and in the abaxial parenchyma of soybean cotyledon tissue is shown in Figure 3.

## Data processing and evaluation

**Figure 4:**
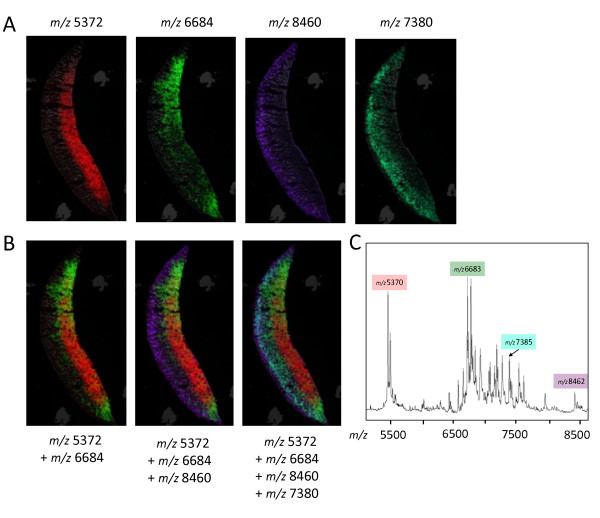
**MALDI-imaging in soybean cotyledons**. (A) MALDI-MSI cross section of a soybean cotyledon. The localisation of 4 distinct *m/z *values is shown. (B) The individual peaks are overlayed in the same MALDI-MSI section. (C) The average mass spectrum of the cross section is shown highlighting the *m/z *values of interest.

Once the data acquisition is complete, individual spectra across the tissue section are aligned and compiled into an imaging map. Using some software packages, spectra processing such as baseline subtraction and smoothing is performed during the data acquisition process. For data acquisition, a range of imaging software packages are available. Some are open-source such as BioMap available from Novartis http://www.maldi-msi.org and ImageJ developed for the NIH http://rsbweb.nih.gov/ij; whereas others are vendor specific and tailored to the instrument and data format produced. In general the software packages are designed to overlay the optical image with the mass spectra acquired. Furthermore software available enables post processing for data analysis. More information on the wide range of software solutions is available from http://www.maldi-msi.org. MALDI-MSI produces very large data sets when obtained at high spatial resolution data. Powerful computer processors and large rapid access memory capacity are therefore required to process these data. Using hierarchical clustering (HC) and principal component analysis (PCA) images can be reconstructed, highlighting peaks as well as regions in the tissue that distinguish sample groups [51,64]. For review of the statistical methods used in MALDI-MSI a comprehensive tutorial is available [65]. The use of serial sections and 3D volume constructions can allow the *in silico *reconstruction of the organ analysed [65]. The layering of data showing the distribution of specific molecules of ions can be used to reconstruct images highlighting spatial features of tissue samples, as shown for soybean cotyledons from our own work in Figure 4.

## Biomarker identification by mass spectrometry

MALDI-MSI leads to the discovery of biomarkers and molecular weight ions of interest; however identification remains a secondary step in the analysis process. Identification can be carried out following protein extraction from the remaining tissue sample; however the most direct approach is to identify proteins directly on the section used for the imaging (Figure 2B). Both strategies have drawbacks: "off-slide" extraction and fragmentation is complicated by the difficulties of matching ions between the extract and the tissue section, whereas "on-tissue" analysis of intact ions is complicated by low ion intensity and the charge state of the ions in the MALDI-ToF. On-tissue digestions with trypsin can be performed while maintaining localisation, however this technique requires optimisation [66]. Furthermore, MALDI-ToF/ToF instruments produce lower fragmentation yields, due to lower energy of the parent ion and the multitude of fragment ions generated during MS/MS. Immonium ions, internal fragments, N-terminal ions or C-terminal ions are commonly observed [66]. Using collision-induced dissociation (CID) or electron-transfer dissociation (ETD) in an ion-trap for fragmentation allows selection of specific precursor ions and furthermore allows acquisition of high resolution and information-rich spectra with significantly reduced chemical noise compared to conventional MALDI-ToF instrumentation [67,68].

Efforts into the development and optimisation of identification strategies of the molecular ions in the profiles imaged are still required and the identification of ions of interest is a bottleneck in MSI. Some of the strategies available are reviewed however to date none have been successful using plant tissue. Trypsin digestion of the tissue and subsequent MALDI-MS/MS analysis of the peptides on-slide has been used successfully for some animal and human tissue samples, particular efforts have been made for formalin-fixed paraffin-embedded (FFPE) tissues [11,64,69,70]. Using in-source decay (ISD) MS during MALDI-MSI has allowed multiple peptides to be sequenced for identification directly on tissue [64,71]. Fewer fragment types are produced in ISD compared to MS/MS, thus the less complex product ions spectra allow easier interpretation and make ISD a good approach for identification of purified intact proteins or peptides without enzymatic digestion [72]. Although this method is not optimal in complex mixtures, Debois *et al*. [71] optimised MALDI-ISD for tissue imaging of porcine eye lens and mouse brain sections. MALDI-MSI using a MALDI-ToF/ToF instrument produced good signal-to-noise MS spectra in the 1000-6000 Da range. Imaging maps could also be produced showing distinct localisation of some ions. Although the fragment spectra are complicated, this research group sequenced a number of sequence tags to use for identification of molecular ions directly on the tissue section. Using HC and PCA analysis for ion selection Bonnel *et al*. [64] were able to identify a number of N-terminus derivatised sequence tags by ISD from FFPE embedded prostate cancer tissue.

Atmospheric pressure ionization (API)-MALDI sources allow MSI analysis, coupled to a Q-ToF or ion-trap MS instrument. API-MALDI-MSI has also been used for imaging of small proteins and peptides and subsequent MS/MS of small molecules and peptides directly on the tissue [18,20,45]. Glucosinolates were identified by CID experiment in the tissue using tandem MS. Similarly, laser spray ionisation (LSI) MS imaging uses laser ablation for high mass compounds on high performance API-MS [68]. The authors compared different imaging MS methods using mouse brain tissue sections. LSI-MS using an Orbitrap Exactive or LTQ Velos (Thermo Scientific) enabled analysis of multiple charged ions directly from the tissue, which could be fragmented and identified using ETD. This approach appears to be an important progress in biomarker identification from tissue sections. Finally, using the data obtained from the imaging experiment, regions of interest can be dissected and digested with trypsin for LC-MS/MS identification.

Off-slide extraction and separation of proteins by electrophoresis for subsequent digestion and peptide identification has also been trialled (Figure 2B). In 2008, Burnum *et al*. [73] studied the changing protein profiles during embryo implantation in mice using MALDI-MSI. Studying the spatio-temporal differences, 50 peaks were found to change due to the presence and location of the embryo. Four proteins were identified using HPLC-separation of intact proteins in a crude extract. The collected fractions were analysed by MALDI-ToF MS and further separated by SDS-PAGE. Bands of approximate *M*r were excised and in-gel digests were identified using LC-MS/MS. More recently, Rauser *et al*. [2] identified a peak at *m/z *8404 that distinguished between HER2-positive and HER2-negative breast cancer tumours. A subsequent section was extracted and proteins separated by high-performance reverse-phase chromatography. Mass-directed fractionation was achieved using the same MALDI-ToF mass analyser and the protein of interest was analysed further using an ETD-ion-trap mass spectrometer. Gustafsson *et al*. [74] analysed tryptic peptides using MALDI-MSI from FFPE brain sections and then extracted the peptide for LC-MS/MS separation and identification. In total 67 potential identifications were reported, which can be followed up by IHC and/or selected reaction monitoring (SRM).

## Conclusions

MALDI-imaging has the potential to provide new insights into the molecular analysis of plants by providing high resolution information on the spatial arrangement of peptides and proteins. It will provide a means of localising differences between plant samples associated with tissue types, development, disease, genetic differences or following genetic manipulation. This review highlights some of the advances made and techniques available, with a special focus on plant tissues. We provide an experimental protocol as a "Beginner's guide" in the additional file 1 However, major challenges lie ahead for the further development of these tools in plants, namely; the need for advances in cryo-sectioning of plant tissues; for further work on matrix types; and the optimisation of matrix concentration and deposition strategies. Better matrix applications are required to allow tissue to tissue comparisons and quantitation. More robust and transferable protocols will allow more reproducibility and therefore quantitative power. This is especially important in plants where limited specific stains or IHC antibodies are currently available to study the spatial distribution of proteins within tissues.

## Competing interests

The authors declare that they have no competing interests.

## Authors' contributions

JG carried out the plant imaging in this manuscript and wrote the first draft of the manuscript. NLT was involved in the imaging studies and participated in the design of the review and the imaging protocols and in the figure production. AHM was involved in the design of the review and the imaging studies, and helped to draft the manuscript. All authors read and approved the final manuscript.

## Supplementary Material

Additional file 1 **Supplementary Protocol: A Beginner's Guide to MALDI-MS Imaging of Proteins in Plant Tissue**. A step-by-step experimental protocol for MALDI-MS imaging of plant tissue. The protocol starts with tissue collection, preparation and storage; covers section preparation for MALDI-imaging and analysis and concludes with data processing. The protocol further includes tips for various steps and optional steps. Click here for file
