# Supplementary material for: Matrix-assisted laser desorption/ionisation mass spectrometry imaging and its development for plant protein imaging
Source: Plant Methods. 2011 Jul 5;7:21. doi: 10.1186/1746-4811-7-21 (PMC3141805; doi:10.1186/1746-4811-7-21)
Supplement: Additional file 1 — Supplementary Protocol: A Beginner's Guide to MALDI-MS Imaging of Proteins in Plant Tissue. A step-by-step experimental protocol for MALDI-MS imaging of plant tissue. The protocol starts with tissue collection, preparation and storage; covers section preparation for MALDI-imaging and analysis and concludes with data processing. The protocol further includes tips for various steps and optional steps. [file 1746-4811-7-21-S1.DOC]

**Grassl *et al.* Additional File 1**

**Supplementary Protocol: A Beginner’s Guide to MALDI-MS Imaging of Proteins in Plant Tissue**

*This protocol was optimised using soybean cotyledons. Leaves from soybean, Arabidopsis and wheat have also been imaged using this technique.*

**Sample preparation**

1. Plant tissue should be prepared immediately after harvesting in order to avoid proteolysis.
2. Optionally plant tissue may be frozen right away, embedded in gelatine, soaked in sucrose or another embedding medium.
3. Leaves are best frozen flat at -80ºC.
4. Sucrose soaking: Prepare 20% sucrose solution in double deionised (ddi) water. Immerse tissue and apply vacuum for 2 min.
5. Gelatine embedding: Prepare 5% gelatine in ddi water, embed using aluminium foil moulds. Allow gelatine to cool down before embedding.

Freeze plant tissue immediately by placing it on cooled metal (in dry-ice) or placing plant tissue sealed in plastic at -80^o^C.

**Cryosectioning and slide preparation**

*MALDI-MSI can be performed in conductively-coated glass microscope slides as well as steel or gold-plates. The use of glass slides has the advantage of allowing microscopy in order to identify regions of interest in the tissue as well as post MALDI-MSI staining.*

1. The plant tissue is attached using a small amount of tissue freezing medium.
2. Cut a series of adjacent sections through the tissue at 12 μm thickness on a cryostat and thaw-mount them onto coated microscope slides. Collect sections with a spacing appropriate for the tissue structure, for example a leaf will yield a much smaller number of sections when cut paradermally than abaxially. Leave a space in the middle of the slide, in order to fit a cover slip.
3. Warm the slide using the palm of the hand inside the cryotome.
4. Remove warmed slide and place into a vacuum dessicator for a minimum of 30 min.

*We commonly cut sections at 10-15 μm, depending on the type of tissue. Thicker sections are not recommended for MALDI-MSI because of the increased risk of tissue distortion during drying.*

1. To fix tissue and remove salts that interfere with the MALDI process, wash sections 2x for 30 seconds in ice cold 70% 2-propanol, followed by one wash in 95% 2-propanol for 15 seconds. Drain excess liquid and dry in a vacuum dessicator for 10 min.

*Freezing the washing solvent allows for better fixing of soluble protein. Other organic solvents have been used as well as longer washing times up to 2 minutes. This is tissue, species and probably protein dependent. The use of xylene has been shown to be beneficial for the analysis of lipids.*

*Optional.* Freeze the dried sections at –20 °C. When ready for use, slowly thaw sections by placing the sealed storage container at 4 °C for 10 min followed by 10 min at room temperature in a vacuum dessicator.

**Matrix application**

*The choice of matrix depends on the samples to be analysed, small molecules and lipids are not covered here.*

*Protein analyses (>3,000 Da):*

- *10 mg/ml sinapinic acid (SA) in 60% acetonitrile and 0.2% TFA in water.*

*Peptides (<3,000 Da):*

- *10 mg/ml α-Cyano-4-hydroxycinnamic acid (CHCA)in 50% acetonitrile and 0.2% TFA in water*

*or*

- *30 mg/ml 2,5-dihydroxybenzoic acid (DHB) in 50% methanol and 0.2% TFA in water. DHB is also used for analysis of lipids and carbohydrates.*

1. Mark sections with white correction fluid (i.e. Tipp-Ex or Liquid Paper) or glass cutter on the slide for orientation.
2. Record an image of the sections keeping the slide in the correct orientation.
3. The matrix needs to be completely dissolved in solution to obtain a homogeneous layer across the entire section.
4. The matrix can be applied dry, using a paintbrush or wet using a spray nebuliser or a spotter, depending on the application. When using a spay nebuliser allow enough drying time between applications in order to avoid migration of analytes. The matrix needs to be completely dry before analysis.

*Wetter applications produced better spectra but localisation of the analytes is lost. Dryer applications show improved localisation but les peak intensity in the spectra.*

*Optional: the slides can be stored at -80 ^o^C once completely dry.*

**Mass Spectrometry using MALDI-ToF instruments**

*The image or micrograph of the section is loaded and aligned with the MSI analysis raster. Correct alignment is important for imaging analysis.*

1. For proteins, analyse sinapinic acid matrix-coated sections on a MALDI-TOF instrument operating in linear mode (currently instruments of choice are: UltrafleXtreme, Ultraflex and Autoflex MALDI-TOFs or MALDI-TOF/TOFs (Bruker Daltronics) or 4800 MALDI-TOF/TOF (AB Sciex). For peptide analysis, CHCA or DHB matrix–coated sections are analysed on a MALDI-TOF operating in reflectron mode.

*The shot number and the size of the laser as well as size of the spot can be optimised for each sample. By shooting an area until there is no analyte left and then moving position the size of the raster needed can be optimised. If there is fresh analyte at the new position then the pixel size is optimal, if not, then the size of the raster has to be increased. Too few laser shots give poor signal-to-noise ratios and too many lead to unwanted noise accumulation after the analyte has been ablated.*

1. Spectra are accumulated in this way using software specific to each vendor instrument, using the appropriate raster size. We recommend baseline subtractions be included as part of the method. The complete images can be opened in a variety of software packages (e.g. FlexImaging ([Bruker Daltonics](http://www.google.com.au/url?sa=t&source=web&cd=8&ved=0CFgQFjAH&url=http%3A%2F%2Fwww.bdal.com%2Fsolutions%2Fclinical%2Ftissue-imaging%2Foverview.html&ei=oWGcTZj2EJHSuwPfp6XSBg&usg=AFQjCNHyZtQjHo7OI5y95FiS7qr-L5o-LA)), MMSIT/4000 series imaging (AB Sciex) or Biomap (Novartis)). For statistical analysis, exported spectra can be opened and analysed using other specialised software (e.g. ClinProtTools ([Bruker Daltonics](http://www.google.com.au/url?sa=t&source=web&cd=8&ved=0CFgQFjAH&url=http%3A%2F%2Fwww.bdal.com%2Fsolutions%2Fclinical%2Ftissue-imaging%2Foverview.html&ei=oWGcTZj2EJHSuwPfp6XSBg&usg=AFQjCNHyZtQjHo7OI5y95FiS7qr-L5o-LA)) or TissueView (AB Sciex))

**Biomarker Identification**

*This is still an emerging area of primary research, the success of different approaches depends on the ions of interest, their relative abundance and the availability of tissue samples beyond the image sections. Details of successful approaches to date are outlined in the review text.*
